# Supplementary material for: Polyketide synthase gene clusters in the ericoid mycorrhizal fungus Oidiodendron maius: genome-wide prediction, expression profiling, and functional characterization of knockout mutants
Source: Front Plant Sci. 2026 Sep 16;17:1924430. doi: 10.3389/fpls.2026.1924430 (PMC13623903; doi:10.3389/fpls.2026.1924430)
Supplement: Supplementary file 1 [file SupplementaryFile1.zip › Supplementary Materials/Supplementary Figures_1-4.docx]

**Supplementary Figures**

**Figure S1**. Distributions of normalised read counts for two sets of RNA-seq data. **Myc**: Mycorrhizal roots. **FLM**: Free living mycelia. **Myc.Cd**: Mycorrhizal roots with cadmium treatment. **FLM.Cd**: Free living mycelia with cadmium treatment. See details of genes with log2 transformed reads and differential transcription. See details of genes with log2 transformed reads and differential transcription in Tables S10, S12 and S13.

(a)

(b)

**Figure S2.** **Structure of the *OmPKS197601* disruption vector. (a)** The plasmid pCAMBIA0380 constitutes the backbone of pCAMBIA0380-Hyg-PKS-KO; the diagram shows the *E. coli* and the *A. tumefaciens* origins of replication (Rep_Origin_1 and 2), the kanamycin resistance gene (Kan_R1), the selection marker for hygromycin resistance (hph-cassette) and the 5’ upstream and 3’ downstream OmPKS197601 flanking regions (1040bp each), that drive the homologous recombination to the desired genome site. (b) Scheme of the predicted homology-driven recombination of the hph-cassette in the *O. maius* genome (left), leading to the disruption of the OmPKS197601 gene (right).

**Figure S3. Integrated multi-omics landscape of entire scaffolds**. Scaffolds 1 and 3 are presented. Gene clusters are depicted by grey vertical bars. Top panel: Up- and down-regulation of genes. The size of circles represents differential transcription levels in log2 (RNA-Seq data; Martino et al., 2018). Middle panel: Density of all genes (grey) and genes encoding secreted proteins in colour located in the scaffold (grey bar). Bottom panel: Density of total and individual transposable element families.

**Figure S4:** Root colonization by *O. maius* WT and the three *OmPKS197601*-null mutants. The number of coils counted in the roots of colonized *V. myrtillus* plantlets has been normalized by the length of the considered root. The line that divides the box represents the median of the data, the empty round point represents the mean, the small black points represent the single data, the points with red line represent the outliers, the whiskers represent the highest and lowest values, excluding outliers. Adjusted p-values from the pairwise Wilcoxon tests are reported. ns= not significant.
